# Effectiveness of the BNT162b2 mRNA COVID-19 vaccine in children under 5 years

**DOI:** 10.1172/JCI173329

**Published:** 2023-11-01

**Authors:** Christoph Strumann, Otavio Ranzani, Jeanne Moor, Reinhard Berner, Nicole Töpfner, Cho-Ming Chao, Matthias B. Moor

**Affiliations:** 1 Institute of Family Medicine, University Hospital Schleswig-Holstein, Campus Luebeck, Luebeck, Germany.; 2 Barcelona Institute for Global Health, ISGlobal, Universitat Pompeu Fabra, Barcelona, Spain.; 3 Pulmonary Division, Heart Institute (InCor), Hospital das Clínicas da Faculdade de Medicina da Universidade de São Paulo, São Paulo, Brazil.; 4 Department of General Internal Medicine, Inselspital University Hospital Bern, Bern, Switzerland.; 5 Institute of Primary Health Care, University of Bern, Bern, Switzerland.; 6 Department of Pediatrics, University Hospital and Medical Faculty Carl Gustav Carus, Technische Universität Dresden, Dresden, Germany.; 7 University Children’s Hospital, University Medical Center Rostock, University of Rostock, Rostock, Germany.; 8 Department of Pediatrics, Helios University Medical Center, Witten/Herdecke University, Heusnerstr, Wuppertal, Germany.; 9 Cardio-Pulmonary Institute, Universities of Giessen and Marburg Lung Center, German Center for Lung Research, Justus-Liebig University Giessen, Giessen, Germany.; 10 Division of Renal Medicine, Department of Clinical Science, Intervention and Technology and; 11 Division of Pathology, Department of Laboratory Medicine, Karolinska Institutet, Stockholm, Sweden.; 12 Department of Nephrology and Hypertension, Inselspital University Hospital Bern, Bern, Switzerland.

**Keywords:** Infectious disease, Vaccines, Epidemiology

**To the Editor:** Despite approval of the BNT162b2 mRNA vaccine (Pfizer/BioNTech vaccine Comirnaty) for children aged 6 months to 4 years by the European Medicines Agency (EMA) and the Federal Drug Administration (FDA) in 2022, little data on BNT162b2 vaccine effectiveness (VE) are available for this age group. We have retrospectively described the safety of BNT162b2 administered off-label in children younger than 5 years in Germany (1). Using data from this authentication-based retrospective survey obtained between April 14, 2022, and May 9, 2022 (1), we here report VE of BNT162b2 during an Omicron BA.1-2–dominant period.

We analyzed 4,615 children, aged 2.8 ± 1.2 years (mean ± SD), who received their first dose of BNT162b2 on January 1, 2022, or thereafter (Supplemental Table 1 and Supplemental Figure 1; supplemental material available online with this article; https://doi.org/10.1172/JCI173329DS1). We used Cox regression to estimate relative VE of 2 BNT162b2 doses, as indicated in the Supplemental Methods. We used the period between the first and second vaccine dose as the reference period (24.8 ± 0.6 days) and the period ≥7 days after dose 2 to before dose 3 as the postvaccination period (59.5 ± 23.6 days). By this approach, the relative HR of SARS-CoV-2 infections was calculated after administration of 2 versus 1 dose of BNT162b2, and this was transformed to VE by the calculation VE = 100 × (1 – HR).

Table 1 shows that VE was substantial for SARS-CoV-2 infections, symptomatic SARS-CoV-2 infections, and SARS-CoV-2 infections leading to medication use. Differences in dosage of BNT162b2 yielded no change in VE. A sensitivity analysis assessed the geographic and potential sex differences in VE by stratification (Supplemental Table 2). The HR of each model parameter is shown in Supplemental Table 3.

The present analysis showed that, in comparison with 1 dose of BNT162b2 alone, children receiving a second dose of BNT162b2 had a substantially lower risk for being diagnosed with a SARS-CoV-2 infection or experiencing a SARS-CoV-2 infection leading to symptoms or medication use. The present data are well in line with those from an emerging report on the US population, where a VE of 54.2% (95% CI, 45.8–61.2) was reported for previously uninfected children receiving 2 doses of BNT162b2 compared with unvaccinated children (2). The current data have some limitations. First, children are rarely tested for SARS-CoV-2, and medical attention is not often sought for their SARS-CoV-2 symptoms. However, this study coincided with (a) a time when mandatory school/institution testing for SARS-CoV-2 was common in Germany, (b) substantial vaccination efforts and campaigns, and (c) the Omicron BA.1 and BA.2 waves, allowing an unusual window of opportunity for study of many recently vaccinated and previously uninfected individuals in this sensitive age group, experiencing symptomatic or asymptomatic infections. Next, the assessed vaccination strategy of BNT162b2 was not the one approved by EMA and FDA; our strategy consisted of 2, instead of 3, administrations of BNT162b2, at dosages higher than 3 μg in most participants. Furthermore, the reported VE does not necessarily correspond to the currently circulating SARS-CoV-2 variants. Finally, the present data are retrospective and await confirmation by prospective and randomized studies. In conclusion, this study offers early, industry-independent insight into the potential VE of the BNT162b2 vaccine in children aged below 5 years, at a time when few immunogenicity or VE data are available (2, 3).

## Supplementary Material

Supplemental data

Supporting data values

## Figures and Tables

**Table 1:**
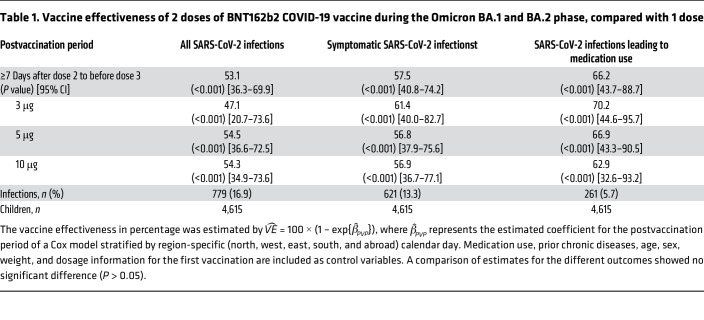
Vaccine effectiveness of 2 doses of BNT162b2 COVID-19 vaccine during the Omicron BA.1 and BA.2 phase, compared with 1 dose

## Version 1 09/05/2023

In-Press Preview

## Version 2 11/01/2023

Electronic publication
