# Supplementary material for: Effectiveness of the BNT162b2 mRNA COVID-19 vaccine in children under 5 years
Source: J Clin Invest. 2023 Nov 1;133(21):e173329. doi: 10.1172/JCI173329 (PMC10617758; doi:10.1172/JCI173329)
Supplement: Supplemental data [file jci-133-173329-s070.pdf]

Vaccine effectiveness of BNT162b2 mRNA Covid-19 vaccine in children under 5 years

Supplementary appendix

Table of contents

Supplementary methods and results... p.2

Author contributions p.4

Conflicts of interest p.5

Acknowledgements p.5

Data availability p.6

Figure S1 .... p.7

Table S1 .... p.8

Table S2 .... p.9

Table S3 .... p.10

Supplementary references p.12

Supplementary methods and results

Study approval

The CoVacU5 study protocol was approved by the Ethics Committee of University of Rostock, Germany (study ID: A2022-0065).

Population and vaccinations:

Design of the authentication-based CoVacU5 survey, recruitment of participants and the authentication procedure have previously been described (1). The study was registered in the German Clinical Trials Register (ID: DRKS00028759) and was conducted adhering to the Declaration of Helsinki. The authors vouch for the fidelity to the approved study protocol. Legal guardians of the studied children gave informed consent to participate in the CoVacU5 study. The vaccines themselves were administered independently from the CoVacU5 study. Accordingly, the legal guardians of the studied children had previously given informed consent to the vaccinations. The physicians responsible for the vaccinations had informed them about potential side effects and about the liability of off-label medicine use under the German law. Independently from this study, the physicians are also required to report all unexpected or severe side effects experienced by the children to the German authorities.

In the present analysis, we used the authenticated participants of the CoVacU5 dataset (1) and excluded those observations with vaccinations prior to January 1st 2022 (n=2045) and with prior SARS-CoV-2 infections (n=113). Further, n=331 datasets with missing information about the vaccination date and n=26 datasets with missing information about the weight were excluded from the analysis resulting in a sample of n=4615 observations (Figure S1, Table S1).

Definition of follow-up periods

We considered the observation days of the period between first and 1 day before the second BNT162b2 administration as reference period to compare with (2,3). We followed the children up to the first positive PCR or antigen-test for SARS-CoV-2 or until 09th of May 2022, whichever came first and censored individuals upon a receipt of third dose of BNT162b2 vaccine. We defined all observation days from day 7 after the second BNT162b2 administration until the day before receiving a third dose of BNT162b2 as the "vaccinated follow-up" period.

Outcomes

Co-primary outcomes of the study were all SARS-CoV-2 infections, symptomatic SARS-CoV-2 infections, or SARS-CoV-2 infections leading to medication use. Secondary outcomes were the different dosage strata of BNT162b2 and the sex-stratified analysis of vaccination effectiveness (VE).

Statistical analysis

All eligible participants were included in the present analysis without sample size estimations. Statistical analysis was performed using STATA version 15 according to the statistical analysis plan available in the supplementary appendix. Two-tailed p values of <0.05 were considered statistically significant. VE was estimated as 100*(1-HR) using Cox regression models with time-varying exposure using vaccination status as a covariate that is not stable but time-dependent. VE was calculated as $\widehat{VE} = 100 \times (1 - \exp\{\hat{\beta}_{PVP}\})$, where $\hat{\beta}_{PVP}$ is the estimated coefficient for the Post-vaccination period of a Cox model stratified by region specific calendar day. By this approach, all individuals at risk in that region on that day contribute to the hazard ratio depending on the number of vaccines they have previously received. The region stratification was chosen to control for potentially different baseline SARS-CoV-2 infection rates over time, and it included 5 regions (northern, western, eastern, southern region of

Germany, and abroad). Similarly, inclusion of calendar time has shown to substantially decrease bias in Cox models of VE (4). Confounders included in the models were medication use, prior chronic diseases, age, sex, weight. Further, we included dummy variables to control for the more precise geolocation on the federal state level. Finally, since the population included children vaccinated with different doses of BNT162b2, we investigated the effects of dosage in an interaction term together with the follow-up period. We tested the estimates of outcomes for differences using z=d/SE(d) where d is the difference between estimates and SE(d) their common standard error (5). As a sensitivity analysis, data were analyzed with stratification for geolocalization in 5 large regions or for sex (Table S2). As another sensitivity analysis, patient ID was entered in the model as a clustering term, with unchanged results (not shown). Table S3 shows the hazard of each of the included model parameters. Additional robustness checks were made by testing the proportional hazards assumption of all variables in the models, and by multiple imputation of weight and vaccination dosage in the dataset of 4979 children (data not shown). No adjustments were made for multiplicity.

Author Contributions:

Dr Strumann had full access to all of the data in the study and takes responsibility for the integrity of the data and the accuracy of the data analysis. Drs M.B. Moor and Chao are co–senior authors.

Concept and design: Strumann, Ranzani, J. Moor, Chao, M.B. Moor.

Acquisition, analysis, or interpretation of data: Strumann, Ranzani, J. Moor, Berner, Toepfner, Chao, M.B. Moor.

Drafting of the manuscript: M.B. Moor.

Critical revision of the manuscript for important intellectual content: Strumann, Ranzani, J. Moor, Berner, Toepfner, Chao, M.B. Moor.

Statistical analysis: Strumann.

Administrative, technical, or material support: Toepfner, Berner, Chao.

Supervision: Chao, Moor.

Conflict of Interest Disclosures:

The authors declare no other conflicts of interest.

Funding:

OR is supported by the Sara Borrell Fellowship from the Instituto de Salud Carlos III (CD19/00110). JM received funding from the Gottfried & Julia Bangerter-Rhyner Foundation. NT and RB were supported by the coverCHILD project of the German Network University Medicine. RB was also supported by the Federal Ministry of Education and Research (BMBF), Germany, and the Saxon State Ministry for Science and the Arts (SMWK), Federal State of Saxony, Germany. CMC was supported by University Medical Center Rostock. MBM is supported by the Swiss National Science Foundation (grant no. 214187).

Acknowledgements:

The authors wish to thank all participating vaccination centers and respondents. The authors acknowledge the contributions to database design by Denisa Drinka, MD and Maximilian Jorczyk, MD (Technische Universität Dresden, Germany) and for participant recruitment by Wolfgang von Meißner, MD (Hausärzte am Spritzenhaus, Baiersbronn, Germany), David Stuppe, MD (University Medical Center, Rostock, Germany), Johannes Püschel, MD (Hausarztzentrum, Greven, Germany), Melanie Liss, MD (Praxis für Kinder- und

Jugendmedizin, Düsseldorf, Germany), Emilie von Poblotzki (Praxis die Kinderärzte, München, Germany), Anke Böhnke, MD (Frauenarztpraxis, Berlin, Germany), Armin Philipp, MD (Hautarztpraxis, Stuttgart, Germany), Georg Hillebrand, MD (Klinik für Kinder- und Jugendmedizin, Itzehoe, Germany), Lisa Degener, MD (Hausärztliche Gemeinschaftspraxis, Altenberge, Germany), and Lisa Schneider, MD (Gemeinschaftspraxis Dres Albrecht/Winkler, Laupheim, Germany). We acknowledge the nonprofit assistance for participant recruitment provided by the Bildung Aber Sicher and U12Schutz initiatives. None were compensated for their work on the CoVacU5 study.

Data availability

The raw data are available from the corresponding author on request, provided that a positive evaluation by the pertinent ethics committee is available and German data protection laws are followed. Data used to generate Supplemental Figure B and Supplemental Table 1 are added as supporting values table.

Supplementary figure

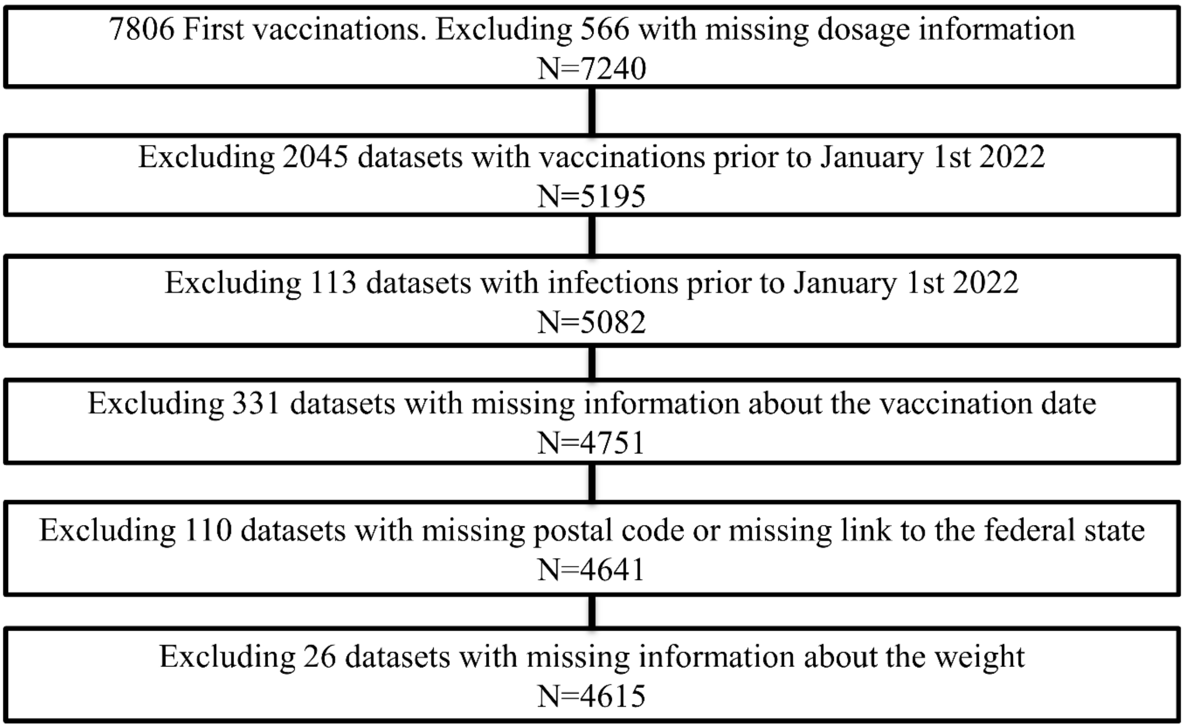

Figure S1. Flow-chart of study participants.

Supplementary tables

Table S1. Characteristics of Children

**Characteristic**	**Total n=4979**	**analyzed sample n=4615**
Sex		
Female	2439 (49.0)	2262 (49.0)
Male	2539 (51.0)	2352 (51.0)
Divers	1 (0.0)	1 (0.0)
Age, median (IQR), y	3 (2-4)	3 (2-4)
Weight, median (IQR), kg	14 (12-17)	14 (12-16.5)
Dosage at first vaccination, µg		
3	1772 (24.5)	833 (18.0)
5	3120 (43.1)	2162 (46.8)
10	2348 (32.4)	1620 (35.1)
Long-term medication	239 (4.8)	217 (4.7)
Comorbidities (yes)	409 (8.2)	376 (8.1)
Regional variables		
Abroad	267 (5.4)	243 (5.3)
Schleswig Holstein	276 (5.5)	234 (5.1)
Hamburg	201 (4.0)	180 (3.9)
Lower Saxony	562 (11.3)	539 (11.7)
Bremen	50 (1.0)	49 (1.1)
North Rhine-Westphalia	1194 (24.0)	1085 (23.5)
Hesse	313 (6.3)	281 (6.1)
Rhineland Palatinate	269 (5.4)	256 (5.5)
Baden-Württemberg	790 (15.9)	766 (16.6)
Bavaria	649 (13.0)	591 (12.8)
Saarland	35 (0.7)	32 (0.7)
Berlin	343 (6.9)	329 (7.1)
Brandenburg	96 (1.9)	93 (2.0)
Mecklenburg Western Pomerania	33 (0.7)	31 (0.7)
Saxony	108 (2.2)	101 (2.2)
Saxony-Anhalt	32 (0.6)	29 (0.6)
Thuringia	41 (0.8)	38 (0.8)

The analyzed sample was used for the main analyses. The total population included missing data in weight and/or vaccine doasage and was used for multiple imputation. Brackets indicate % unless stated otherwise.

Table S2: Vaccination effectiveness of BNT162b2 stratified per region or sex

	**All SARS-CoV-2 infections**<br>% (p-value)<br>[95%-CI]	**Symptomatic SARS-CoV-2 infections**<br>% (p-value)<br>[95%-CI]	**SARS-CoV-2 infections leading to medication use**<br>% (p-value)<br>[95%-CI]
Region			
**Northern**	35.6<br>(0.232)<br>[-0.227;0.939]	35.6<br>(0.268)<br>[-27.5;98.8]	56.3<br>(0.100)<br>[-10.8;123.3]
Infections, n/N (%)	145/894(16.2)	119/868(13.7)	39/788(4.9)
**Eastern**	53.9<br>(0.005)<br>[16.4;91.4]	60.6<br>(<0.001)<br>[26.4;94.8]	86.4<br>(<0.001)<br>[65.6;107.2]
Infections, n/N (%)	96/565(17.0)	77/546(14.1)	33/501(6.6)
**Southern**	56.1<br>(<0.001)<br>[24.3;88.0]	73.2<br>(0.000)<br>[53.7;92.7]	68.7<br>(0.025)<br>[8.8;128.5]
Infections, n/N (%)	228/1319(17.3)	175/1266(13.8)	67/1158(5.8)
**Western**	63.0<br>(<0.001)<br>[43.5;82.6]	57.1<br>(<0.001)<br>[30.9;83.3]	61.2<br>(<0.001)<br>[23.8;98.5]
Infections, n/N (%)	269/1594(16.9)	213/1539(13.8)	98/1423(6.9)
**Abroad**	-23.8<br>(0.893)<br>[-371.3;323.6]	-3.5<br>(0.981)<br>[-294.5;287.5]	24.3<br>(0.768)<br>[-137.0;185.5]
Infections, n/N (%)	42/243(17.3)	37/238(15.5)	24/225(10.7)
Sex			
**females**	49.0<br>(<0.001)<br>[22.7;75.4]	58.2<br>(<0.001)<br>[35.8;80.7]	55.0<br>(0.027)<br>[6.4;103.7]
Infections n/N (%)	354/2262(15.6)	284/2192(13.0)	113/2020(5.6)
**males**	54.3<br>(<0.001)<br>[31.6;77.0]	54.0<br>(<0.001)<br>[27.0;81.0]	61.2<br>(<0.001)<br>[25.3;97.1]
Infections n/N (%)	425/2352(18.1)	337/2264(14.9)	148/2074(7.1)

N is the number of children used in the estimation; n is the number children that have an infection in the respective region. The post-vaccination period of ≥7 Days after Dose 2 to before Dose 3 was compared with the period between Dose 1 and Dose 2 as a reference. Region refers to residence within Germany.

Table S3: Estimated hazard ratios

**Variable**	**M1**	**M2**	**M3**	**M4**
Follow-Up (reference FU1: Dose 1 to before Dose 2)				
FU2: Dose 2 to <7 days after Dose 2	0.434***	0.429***	0.429***	
FU3: >=7 Days after Dose 2 to before Dose 3	0.467***	0.464***	0.469***	
Dosage specific FU-Effects				
FU2 X Dosage 3µg				0.669
FU2 X Dosage 5µg				0.383***
FU2 X Dosage 10µg				0.385***
FU3 X Dosage 3µg				0.529**
FU3 X Dosage 5µg				0.455***
FU3 X Dosage 10µg				0.457***
Controls				
dosage at first vaccination (ref. 3µg)				
5µg		0.904	0.894	1.028
10µg		0.946	0.941	1.077
age (ref. 0.5)				
1		1.165	1.168	1.163
1.5		0.941	0.932	0.933
2		1.591*	1.588*	1.579*
2.5		1.700**	1.667**	1.654*
3		1.966**	1.952**	1.937**
3.5		2.005**	1.989**	1.978**
4		1.976**	1.982**	1.970**
4.5		2.485***	2.432***	2.414***
female		0.886*	0.884*	0.885*
weight		0.99	0.991	0.992
Long-term medication		1.442*	1.432*	1.437*
Comorbidities (yes)		1.084	1.091	1.089
regional variables *(ref. abroad)*				
Schleswig Holstein			1.468	1.473
Hamburg			1.213	1.219
Lower Saxony			1.296	1.299
Bremen			0.827	0.829
North Rhine-Westphalia			1.523	1.533
Hesse			1.248	1.253
Rhineland Palatinate			1.326	1.336
Baden-Württemberg			0.455	0.454
Bavaria			0.411	0.41
Saarland			1.892	1.899
Berlin			1.399	1.362
Brandenburg			1.601	1.571
Mecklenburg Western Pomerania			2.105**	2.069**
Saxony			1.284	1.241
Saxony-Anhalt			1.853	1.779
Thuringia			1.673	1.601
LOGLIKE	-2372.0	-2341.3	-2334.8	-2333.8
AIC	4748.1	4714.6	4733.7	4739.6
BIC	4769.8	4888.6	5081.6	5131.1

* p<.1; **p<.05; *** p<.01; observations from 4615 children used in the estimation; stratified by region specific (north, west, east, south, and abroad) calendar day; LOGLIKE: Loglikelihood value; AIC: Akaike information criterion; BIC: Bayesian information criterion

Supplementary references

1. Toepfner N, von Meißner WCG, Strumann C, Drinka D, Stuppe D, Jorczyk M, et al. Comparative Safety of the BNT162b2 Messenger RNA COVID-19 Vaccine vs Other Approved Vaccines in Children Younger Than 5 Years. JAMA Netw Open. 2022 Oct 18;5(10):e2237140–e2237140.

2. Fabiani M, Puopolo M, Morciano C, Spuri M, Spila Alegiani S, Filia A, et al. Effectiveness of mRNA vaccines and waning of protection against SARS-CoV-2 infection and severe covid-19 during predominant circulation of the delta variant in Italy: retrospective cohort study. BMJ. 2022 Feb 10;376:e069052.

3. Cerqueira-Silva T, Oliveira V de A, Boaventura VS, Pescarini JM, Júnior JB, Machado TM, et al. Influence of age on the effectiveness and duration of protection of Vaxzevria and CoronaVac vaccines: A population-based study. Lancet Reg Health Am. 2022 Feb;6:100154.

4. Lund LC, Støvring H, Pottegård A, Andersen M, Hallas J. Cox regression using a calendar time scale was unbiased in simulations of COVID-19 vaccine effectiveness & safety. J Clin Epidemiol. 2023 Apr;156:127–36.

5. Altman DG, Bland JM. Interaction revisited: the difference between two estimates. BMJ. 2003 Jan 25;326(7382):219.
